# From Sentiment to Signal: Narrative–Physiology Discordance as a Testable Target for Artificial Intelligence in Critical Care

**DOI:** 10.3390/medsci14050595

**Published:** 2026-09-21

**Authors:** Ignacio Martin-Loeches, Hongliu Cai

**Affiliations:** 1 Department of Intensive Care Medicine, Multidisciplinary Intensive Care Research Organization (MICRO), St James’s Hospital, St James’s Street, D08 NHY1 Dublin, Ireland; 2 School of Medicine, Trinity College Dublin, D02 R590 Dublin, Ireland; 3 Trinity Centre for Biomedical Engineering (TCBE), Trinity College Dublin, D02 R590 Dublin, Ireland; 4 Department of Critical Care Medicine, The First Affiliated Hospital, Zhejiang University School of Medicine, Hangzhou 310003, China

**Keywords:** critical care, clinical sentiment analysis, natural language processing, clinical deterioration, clinical prediction model, calibration, alert fatigue, electronic health records, sepsis, multimodal artificial intelligence

## Abstract

Intensive care units generate dense physiological, laboratory, imaging, microbiological and treatment data, yet much of the clinical reasoning that drives decisions is recorded only in free text. Over the past decade, clinical sentiment analysis has repeatedly shown that the affective tone of nursing and medical notes is associated with mortality. The incremental discrimination over established severity scores has nevertheless been small, of the order of 0.01 in the area under the receiver operating characteristic curve in the largest published intensive care cohort, and general-purpose sentiment tools transfer poorly to critical care text. We argue that this plateau reflects a misspecified prediction target rather than a limitation of natural language processing. Mortality at 28 days is not the question the clinician asks at the bedside. We propose that the clinically useful quantity is the narrative–physiology discordance score: the signed difference, expressed on a common calibrated scale, between the short-horizon deterioration risk implied by the documented assessment and the risk implied by time-aligned multimodal data. We specify this quantity formally, fix index time and forecast horizon, and set out the leakage, copy-forward and provenance controls without which retrospective performance is uninterpretable. We then treat alert burden as a design constraint rather than a post hoc observation, deriving the operating threshold from an explicit and context-dependent alert budget, and we outline a staged evaluation pathway aligned with TRIPOD + AI and DECIDE-AI in which discordant cases are adjudicated by clinicians. Until that adjudication has been performed, discordance is a record-level inconsistency that prompts reassessment, not evidence of missed deterioration. Clinical sentiment analysis becomes useful when it stops predicting death and starts flagging disagreement.

## 1. Introduction

The modern intensive care unit (ICU) is routinely described as data rich. Monitors, ventilators and infusion systems record continuous measurements; the electronic health record (EHR) accumulates laboratory results, radiology, microbiology, medication exposure and organ support. Publicly available critical care databases such as MIMIC-IV now expose these streams, together with deidentified free-text notes, to the wider research community [1]. Yet the most integrated account of a patient’s condition on any given day is usually written in ordinary clinical language. Progress notes, handovers, nursing entries and consultation records carry judgements about response to treatment, reversibility, uncertainty and the proportionality of care that appear nowhere in the structured record.

That judgement is not merely descriptive. In a prospective cohort of 303 critically ill patients, ICU physicians predicted six-month mortality with a C statistic of 0.76, and when they reported being confident in their prediction, the C statistic rose to 0.90; adding physician and nurse predictions to a model containing objective clinical variables improved discrimination significantly for in-hospital mortality, six-month mortality and return to original residence [2]. On the ward, nurses’ expressed worry discriminates deterioration with an area under the receiver operating characteristic curve (AUROC) of 0.81 in isolation, and adding worry-related indicators to a vital-sign-based early warning score raises the AUROC from 0.86 to 0.91 [3]. Clinician concern is therefore a real and partially independent signal, but it is expensive to elicit prospectively, it is unevenly acted upon, and nurses frequently report difficulty converting it into a credible escalation [4]. The attraction of clinical sentiment analysis is that this signal is already deposited, unprompted and free of charge, in text that is written anyway.

Severity scores such as APACHE II [5] and the Sequential Organ Failure Assessment (SOFA) [6] remain indispensable for description, benchmarking and risk adjustment, and organ dysfunction quantified by SOFA is embedded in the current consensus definition of sepsis [7]. Their purpose, however, differs from the task the clinician faces during repeated bedside review. A score computed within a fixed window yields a population-level estimate; it cannot represent a patient whose trajectory changes four hours after a therapeutic intervention.

This article does not argue that clinical sentiment analysis is promising. That argument has been made, and the empirical literature that followed it has plateaued. We argue instead that the field has been optimising the wrong target, and we specify a different one that is concrete, falsifiable and directly linked to a decision. Section 2 examines what the published evidence actually delivers and where it stops. Section 3 defines the narrative–physiology discordance score, states what its narrative component is and is not intended to capture, and distinguishes clinically meaningful discordance from the expected differences between two information modalities. Section 4 and Section 5 address the temporal-validity and evaluation requirements without which such a score cannot be believed. Section 6 treats alert burden as an arithmetic design constraint. Section 7 and Section 8 address governance and a staged research agenda.

## 2. What the Existing Evidence Shows, and Where It Stops

### 2.1. Association with Mortality Is Established; Incremental Value Is Not

The foundational observation is reproducible. In 27,477 ICU patients drawn from MIMIC-III, mean sentiment polarity in nursing notes was independently associated with 30-day mortality after adjustment for sex, ICU type and SAPS-II score (adjusted odds ratio 0.46, 95% confidence interval 0.42–0.50), and sentiment quartiles separated Kaplan–Meier survival curves [8]. In 1851 patients with sepsis, sentiment polarity and subjectivity were both inversely associated with in-hospital 28-day mortality in multivariable Cox models [9]. In 631 patients with acute pancreatitis, a model incorporating nursing-note sentiment achieved an AUROC of 0.812, exceeding SOFA (0.732) and SAPS-II (0.792) [10].

The critical number is rarely emphasised. In the largest of these cohorts, adding mean sentiment polarity to an established severity-based model moved the AUROC from 0.8092 to 0.8189 [8]. An increment of roughly one hundredth in discrimination, obtained from a full admission’s worth of nursing text, is not a foundation for a bedside tool. It is the signature of a variable that is real but almost entirely redundant with what SAPS-II already captures. A construct-validity study spanning 793,725 multidisciplinary notes across 41,283 hospitalisations found the same ceiling from the opposite direction: none of six sentiment methods improved early prediction of in-hospital mortality in logistic regression models, agreement between methods was weak despite positive correlation and median daily lexical coverage ranged from only 5.4% to 20.1% [11].

### 2.2. General-Purpose Sentiment Tools Do Not Transfer to Clinical Text

Part of the plateau is a tooling problem, and here the field has made genuine progress. Sentiment models trained on consumer reviews and social media misread clinical prose, in which “no further deterioration” is reassuring and “patient comfortable” may be a statement about end-of-life care. Domain-specific approaches close much of this gap. Applied to 198,944 notes across 52,997 ICU admissions in MIMIC-III with external replication at a second academic centre, a consensus clinical lexicon and a transformer model trained on clinical sentiment labels correlated with clinician-assigned labels at Spearman coefficients of 0.62–0.84, against 0.28–0.46 for general-purpose classifiers; clinical sentiment terms were present in 99% of patient visits and 88% of notes [12].

Until recently, the field lacked a clinician-annotated ground truth against which such models could be judged. That gap has now been addressed: five clinicians annotated ICU notes over fifteen months, with annotators marking the specific text spans that drove their labels, producing the first publicly available ICU-specific clinical sentiment dataset, on which clinical language models reached accuracies up to 82% and significantly outperformed a keyword-based lexicon [13]. The annotation effort required is itself informative. Fifteen months of clinician time to label a single-institution corpus suggests that the availability of clinician-labelled data, rather than model architecture, may currently be the principal constraint on progress in this field.

### 2.3. Text Carries Substantial Non-Redundant Information When the Target Is Right

The pessimistic reading of Section 2.1 and Section 2.2 would be that clinical narrative adds little. That reading is contradicted by studies in which text is used to answer a different question. In a hospital-wide sepsis detection model developed on 218,715 episodes with prospectively expert-validated sepsis cases, 73.0% of the selected predictors were derived from unstructured clinical notes by natural language processing, against 27.0% from structured data; the resulting model achieved an AUROC of 0.95 with 93% sensitivity and 84% specificity, and reduced false positives by 39.6% relative to the best rule-based comparator [14]. Free text was not marginal in that model; it was the majority of the signal.

The contrast is instructive. When narrative is compressed into a single scalar valence and asked to predict death weeks later, it contributes almost nothing beyond SAPS-II. When narrative is mined for clinical content and asked to identify a state that is happening now, it dominates. The problem is not the text. The problem is the target.

## 3. Reframing the Target: Narrative–Physiology Discordance

### 3.1. The Clinical Question

At the bedside, the intensivist does not ask what this patient’s probability of 28-day mortality is. The operative questions are narrower: is this patient better or worse than four hours ago; does the team’s written impression match what the monitor, the pump and the laboratory are saying; and which of the twenty patients on the unit most warrants a second look before the next round. Mortality prediction answers none of these.

Clinical sentiment is best understood not as generic positive or negative language but as a time-stamped, observer-dependent estimate of trajectory. Its distinctive value is therefore comparative. Everything a sentiment model can say about prognosis is largely recoverable from physiology; what a sentiment model can say that physiology cannot is what the documented assessment implies about the trajectory. The quantity of interest is the disagreement between the two.

Four terms recur in what follows and are used in distinct senses. *Clinical sentiment* denotes the affective polarity of clinical text as measured by the methods reviewed in Section 2; it is the construct that the existing literature has optimised. *Documented assessment* denotes the clinical content of a note: the writer’s stated interpretation of trajectory, response to treatment, reversibility and concern. *Documented concern* is the subset of that content that expresses worry about the patient’s course. The *narrative trajectory score* is the calibrated probability estimated from the documented assessment, as specified in Section 3.2. We avoid the phrase *team belief* as an operational term, because the score has no access to what clinicians think, only to what they wrote.

### 3.2. Formal Specification

Both components must be placed on a single, interpretable and calibrated scale before any difference between them is meaningful. We define both as estimated probabilities of the same pre-specified event.

Let *h* be a fixed forecast horizon (for example 6 or 12 h) and *E*(*t*,*h*) a pre-specified deterioration event occurring in the interval (*t*, *t* + *h*]. For each index time *t*, using only data available at *t*, define

*S_phys_*(*t*) = Pr[*E*(*t*,*h*)|structured multimodal data up to *t*]

*S_narr_*(*t*) = Pr[*E*(*t*,*h*)|narrative text up to *t*]

*D*(*t*) = logit *S_phys_*(*t*) − logit *S_narr_*(*t*)

Three features of this specification matter. First, the difference is signed, not absolute. A positive value indicates that measured trajectory is worse than the documented assessment implies; a negative value indicates documented concern in excess of what the structured data show. These are clinically distinct events and must not be collapsed into a magnitude. Second, the difference is taken on the logit scale, so that a discrepancy between 0.02 and 0.10 is not treated as smaller than a discrepancy between 0.50 and 0.58. Third, both components must be independently calibrated before subtraction. Two poorly calibrated models produce a difference that is uninterpretable even if each has acceptable discrimination [15].

The event *E*(*t*,*h*) must be objective and adjudicable, and it must be the same for both components. Candidate definitions include initiation or escalation of vasopressor therapy above a pre-specified dose; transition to invasive mechanical ventilation; initiation of renal replacement therapy; an unplanned ICU-level intervention or emergency procedure; cardiac arrest; and death within the horizon. A composite will usually be required to obtain an adequate event rate at horizons of 6 to 12 h, and its components should be reported separately. One caution applies to any event that is defined by a treatment decision. Documented concern may itself trigger the intervention that then becomes the prediction target, so that the narrative component partly predicts the team’s plan rather than the patient’s trajectory. This circularity flatters the narrative model and biases *D*(*t*) towards negative values. Event definitions anchored in a physiological state that documentation cannot create, such as a sustained fall in mean arterial pressure or a rising lactate, are preferable; where an intervention is retained as an event, a physiological criterion should be required alongside it, and performance should be reported separately for physiologically anchored and intervention-defined events.

A positive discordance is not a diagnosis, and the model must not be permitted to make one. It is a statement about the record, and it admits at least five explanations: genuine deterioration not yet recognised; recognised deterioration not yet documented; documentation lag or copy-forward; an artefact in the structured feed such as a damped arterial line or an erroneous laboratory value; or a model failure. The interface must surface these as competing hypotheses rather than resolving them. Until prospective adjudication (Section 8) has established how often each explanation applies, *D*(*t*) should be regarded as a record-level inconsistency signal whose purpose is to prompt clinical reassessment, not as evidence that the treating team has missed a deterioration.

### 3.3. Construct: Documented Assessment, Not Transcribed Physiology

The narrative trajectory score is intended to estimate the risk implied by the documented assessment, not to recover physiology that happens to have been written in prose. The distinction matters because clinical notes routinely contain measured values: blood pressure, lactate, inspired oxygen fraction, urine output and ventilator settings are copied or dictated into the text. A model given the whole note will learn to read those numbers, and a difference between two estimates of the same physiology is not discordance in the sense we intend. Three measures follow. First, numerical physiological content, and any text auto-populated from the structured record, should be masked or removed before the narrative model is trained, using a pre-specified and reported masking rule; performance should be reported for the masked and the unmasked narrative model, and only the masked model should contribute to *D*(*t*). Second, the information shared between the two components should be measured rather than assumed: the correlation, or mutual information, between *S_narr_*(*t*) and *S_phys_*(*t*), and the incremental contribution of *S_narr_*(*t*) conditional on *S_phys_*(*t*), are required outputs of any development study, and they bound the information that *D*(*t*) can carry. Third, qualitative characterisations of physiology are retained. When a clinician writes that lactate is climbing or that the patient remains hypotensive despite fluids, the decision to record the observation and the direction assigned to it are interpretive acts, and they are the material the score is meant to read. The boundary between transcribed and interpreted physiology is therefore a design decision that each study must state explicitly, and the adjudication proposed in Section 8 is the only way to establish empirically that what remains after masking behaves as a measure of assessment rather than as a noisy copy of the monitor.

Several professional groups document the same patient within a single window, and their assessments may differ. *S_narr_*(*t*) should not average this away. We propose that role-specific narrative scores, at minimum for medical and nursing notes, be estimated and then combined by a pre-specified rule, for example, the most recent note of each role weighted by recency, and that disagreement between roles be reported as a separate quantity. Divergence between nursing worry and medical reassurance is a recognised antecedent of failed escalation [3,4], and it is more useful surfaced than absorbed. Author role and grade appear in Table 1 as display and provenance elements for this reason. They should not be used as model inputs without specific justification: a model that learns to weight consultant notes differently from junior notes is learning institutional hierarchy rather than clinical concern, and any use of role or grade as a predictor should be accompanied by a stratified evaluation of calibration by author role.

### 3.4. Uncertainty, Expected Modality Differences and Clinically Meaningful Discordance

A point estimate of *D*(*t*) is insufficient for alerting, because the uncertainty of the two components varies between patients and over time. Sparse, contradictory or heavily copied documentation widens the uncertainty of *S_narr_*(*t*); missing values, measurement artefacts and infrequent sampling widen that of *S_phys_*(*t*). Each component should therefore be estimated with a prediction interval, obtained for example by ensembling, bootstrap or conformal methods, and an interval for *D*(*t*) derived from both. We propose that alert eligibility be defined on the interval rather than on the point estimate: an alert is generated only when the point estimate of *D*(*t*) lies beyond the threshold set by the alert budget for that direction (Section 6), the interval for *D*(*t*) excludes zero, and the width of that interval is below a pre-specified maximum. Where the width exceeds that maximum the system abstains, and the abstention is displayed and counted (Table 2). Uncertainty thus determines whether an alert is raised, rather than decorating an alert that has already been raised.

Narrative and structured data are different information modalities, and some disagreement between them is expected rather than pathological. The narrative may legitimately contain information that the structured record cannot represent, including bedside gestalt, work of breathing, skin perfusion and the observed response to a treatment; the structured record may contain a laboratory result or a monitoring trend that arrived after the last note was written and has not yet been incorporated into the narrative. The first produces negative discordance that may be entirely appropriate; the second produces positive discordance that will resolve when the next note is authored. Neither is a failure of the record, and a threshold applied to a single value of *D*(*t*) cannot separate them from discordance that matters. We therefore propose that clinically meaningful discordance be defined operationally by three properties. The first is magnitude: a value of *D*(*t*) beyond the budget-derived threshold. The second is persistence: a discordance that survives the next documentation opportunity, or that is accompanied by rapid change in *S_phys_*(*t*) when waiting is not acceptable. The third, during evaluation, is adjudication: classification by clinician review into one of the categories set out in Section 8 (Table 3), of which only some represent information the treating team needs. The time elapsed since the last note is a covariate of the expected difference; it should be displayed with the score and used to define the reference distribution against which a given *D*(*t*) is judged.

## 4. Temporal Validity, Leakage and Documentation Provenance

### 4.1. Outcome-Adjacent Language Is the Dominant Leakage Pathway

The strongest technical threat to retrospective work in this area is temporal leakage. When notes close to discharge are labelled positive and notes close to death are labelled negative, a model can achieve impressive apparent performance by learning documentation produced after the outcome has already become inevitable. Language concerning transition to comfort-focused care, treatment limitation, family meetings, transfer arrangements or bereavement support is not a prediction of death; it is a consequence of a decision already taken. A model that detects it has learned nothing modifiable.

This is not a hypothetical risk in sentiment work specifically, because such language is precisely the language that carries the strongest affective valence. The mitigation is procedural and must be pre-specified: every note contributing to a prediction at index time *t* must be verified to have been authored before *t*, and an explicit outcome-aware audit must remove or flag notes containing decision-adjacent content that post-dates the index time. Sensitivity analyses excluding the final 24 and 48 h of each admission should be reported as standard. Truncation is a blunt instrument, however, and its cost should be acknowledged. For admissions that end in death, the final day or two is also the period in which clinically meaningful discordance is most likely to precede deterioration, so truncation removes true signal together with leakage. Truncated analyses therefore serve to bound the contribution of outcome-adjacent language, not to replace the primary analysis, and the note-level audit described above, which removes decision-adjacent content rather than whole intervals, is the more specific control. Performance should be reported with and without truncation, and a large difference between the two is itself informative.

### 4.2. Copy-Forward and Note Provenance

Sentiment computed on copied text is a measurement of yesterday presented as a measurement of today. This is not a marginal problem. In one prospective inpatient audit, 92.7% of resident progress notes contained copied content before intervention, and 58% of copied notes contained documentation errors [19]. Note that timing compounds the issue: notes composed and signed late in the day rather than during or immediately after rounds delay the availability of narrative content for downstream processing, so that the text an algorithm reads at 16:00 may reflect an assessment made at 09:00 [20].

Any credible study must therefore report timestamped text-similarity metrics between consecutive notes from the same author and across authors, the proportion of each note that is templated or auto-populated, and the interval between the clinical assessment and note signature where the EHR audit log permits. Notes that are substantially duplicated should either be down-weighted or should trigger abstention, and the abstention rate should be reported as a primary operating characteristic rather than buried in supplementary material.

### 4.3. Index-Time Discipline and Dynamic Evaluation

Short admissions create a related problem. Initial notes frequently document severe physiological derangement alongside a confident impression of rapid reversibility, which is exactly the discordance pattern the model is designed to detect, but for a benign reason. A single prediction per admission cannot distinguish these cases. Each model must instead predict a pre-specified state at a pre-specified index time using only information available at that moment, and performance must be evaluated at repeated landmark times across the admission rather than once.

## 5. Evaluation: What Must Be Reported

Discrimination alone is inadequate. A model with a high AUROC but a calibration slope far from unity will systematically overstate or understate risk at the bedside, and calibration remains the most frequently omitted element of prediction-model reporting [15]. Because the discordance score is a difference between two probabilities, miscalibration in either component propagates directly into the quantity clinicians are asked to act on.

The consequences of skipping this are documented. A proprietary sepsis prediction model deployed at hundreds of hospitals was found on external validation across 38,455 hospitalisations to have a hospitalisation-level AUROC of 0.63, to miss 67% of sepsis cases, and to generate alerts in 18% of all hospitalisations [21]. That combination, poor discrimination and a very high alert rate, is the failure mode that any narrative-based surveillance layer risks reproducing, because notes are written continuously and a discordance score can be recomputed indefinitely.

Reporting should follow TRIPOD + AI for model development and validation [16] and DECIDE-AI for early-stage clinical evaluation [17]. The minimum specification we would expect a reviewer to require is set out in Table 2.

## 6. Alert Burden as a Design Constraint, Not a Finding

Alarm fatigue is an established safety problem in critical care. Systematic reviews consistently report that ICU nurses regard alarms as excessive, that they interfere with patient care, and that they erode trust in monitoring systems, with no clear system for managing them [22,23]. Adding a narrative surveillance layer to that environment without an explicit burden constraint would be indefensible.

We therefore propose that the operating threshold be fixed by an alert budget agreed with the clinical team before the model is trained, rather than chosen post hoc from a receiver operating characteristic curve. The arithmetic is simple and should be stated in the protocol. Consider, purely for illustration, a 20-bed ICU in which the discordance score is recomputed six times per day, giving 120 scoring opportunities per day. An alert rate of 5% yields six alerts per day, approximately 2190 per year. If the positive predictive value at that threshold is 20%, roughly five of those six daily alerts will be non-actionable. If the unit agrees that it will tolerate no more than two alerts per day and will accept no fewer than one actionable alert in three, then the alert rate must not exceed 1.7% and the required positive predictive value is at least 0.33. Numbers of that kind, not the AUROC, define whether the model is deployable. The particular values are not recommendations. Two alerts per day and a positive predictive value of 0.33 have no intrinsic meaning; an acceptable burden depends on unit size and staffing, baseline workload, the severity and urgency of the event being predicted, whether the alert interrupts or is delivered passively at a defined decision point, how it is embedded in the workflow and, on the other side of the ledger, the consequence of a missed deterioration. The budget must be defined with the clinical team that will receive the alerts and tested against that team’s workflow before deployment, and it should be expected to differ between units and to be revised as experience accumulates.

This reframing has a practical consequence for model design. It shifts the objective from maximising discrimination to maximising sensitivity subject to a fixed alert rate, which is a different optimisation and typically favours abstention and selective silence over universal scoring. The interface should follow the same principle: escalation rather than continuous interruption, with visible prompts reserved for rapid trajectory change, substantial and sustained discordance, or a defined decision point such as a handover or a planned ventilator weaning trial.

There is evidence that this can work when the human response pathway is designed alongside the model. In a prospective multi-site evaluation of a machine-learning sepsis early warning system covering 590,736 monitored patients, patients whose alert was confirmed by a provider within three hours had an adjusted absolute reduction in in-hospital mortality of 3.3% and an adjusted relative reduction of 18.7% compared with those whose alert was not confirmed within that window [24]. The benefit was tied to a specific, timed human action, not to the existence of the alert.

## 7. Human Factors, Accountability and Regulation

Implementation experience from deployed critical care machine-learning systems is unambiguous about where the effort goes. The integration of a deep-learning sepsis platform into routine care required sustained investment in stakeholder alignment, role definition and frontline training, and the model itself was a minority of the work [25]. Qualitative evaluation of the same deployment found that clinician trust derived from personal experience of the system’s accuracy rather than from any explanation of the model, and that the principal barriers were information flow between professional groups and gaps in understanding of the surrounding workflow rather than the algorithm [26]. A discordance score, which by construction draws attention to a mismatch between the team’s written assessment and the structured record, will encounter these dynamics in an intensified form. It must be introduced as a prompt to review, never as a contradiction of the bedside clinician, and clinicians must be able to confirm, reject or qualify each output, with those responses captured as monitoring data.

Clinical deployment also creates a difficult allocation of responsibility. A clinician who follows an incorrect recommendation remains accountable; a clinician who overrides a warning that later proves correct may equally be questioned. In the European Union, this is now a regulatory matter as well as an ethical one. Under Regulation (EU) 2024/1689, artificial intelligence systems that are, or are safety components of, devices regulated under the Medical Devices Regulation are classified as high risk and are subject to obligations on risk management, data governance, technical documentation, logging, transparency and human oversight, layered on top of existing conformity assessment [27]. Regulatory certification of clinical machine-learning tools in this space is achievable, and a clear regulatory pathway is itself a precondition of clinician and patient trust [28]. The European Medicines Agency has no role in the conformity assessment of medical devices. Software of this kind is placed on the market by CE marking under the Medical Devices Regulation (EU) 2017/745 [29], following conformity assessment by a notified body, and the hospital-wide sepsis detection model described in Section 2.3 [14] obtained CE marking as a class IIa medical device by that route in June 2024, with the certificate issued by an EU notified body [30]. Because class IIa software requires third-party conformity assessment, such a system also meets the criterion under which Regulation (EU) 2024/1689 classifies a device-embedded artificial intelligence system as high risk. The governance requirements follow directly. Every deployed system needs a named clinical owner, a documented intended use, version control, prospective silent evaluation before any interventional use, audit trails, scheduled drift monitoring and a pre-agreed procedure for suspension.

Drift deserves particular emphasis for narrative models. Structured data models drift when case mix, coding practice or treatment protocols change [18]. Narrative models drift additionally when documentation practice changes, and documentation practice is currently changing rapidly with the introduction of ambient scribes and large language model summarisation. A model trained on human-authored notes and applied to machine-generated notes is operating out of distribution in a way that no structured data monitoring will detect. Surveillance of the input text distribution, not only of model outputs, is therefore mandatory. The two components of *D*(*t*) will also drift at different rates and in different directions, because their inputs are governed by different processes. If only one component loses calibration, *D*(*t*) shifts systematically for every patient on the unit although the records themselves have not changed, and a threshold fixed under the original calibration then generates alerts, or suppresses them, for a reason that has nothing to do with the patient. Calibration must therefore be monitored separately for each component against a rolling reference period, with pre-specified recalibration triggers, and a shift in the population distribution of *D*(*t*) in the absence of a corresponding change in event rate should itself be treated as a drift signal that suspends alerting until both components have been re-calibrated.

## 8. A Staged Research Agenda

The path from the current literature to a deployable tool is not a single study. We propose four stages, each with a defined exit criterion (Table 4).

Two design choices should be embedded from Stage 2 onwards. First, the narrative and structured components must be estimated separately for the discordance score to exist at all, because a jointly trained model cannot produce the decomposable difference that is the entire purpose of the exercise. This is a requirement of the framework, not a claim that late fusion is generally preferable for multimodal prediction: the prevailing direction in multimodal biomedical artificial intelligence favours early or intermediate fusion for maximal predictive performance [31], and it may well be right for the prediction task. Whether decomposability costs discrimination, and how much, is an empirical question, so a jointly trained multimodal model should be included as a comparator at every stage (Table 2) and the discrimination forgone for interpretability reported rather than assumed. Second, subgroup analysis should be pre-specified by clinical phenotype rather than only by demographic strata. Critical illness syndromes contain robust, reproducible subphenotypes with different trajectories and treatment responses [32], and there is no reason to assume that the relationship between documented assessment and measured trajectory is constant across them.

The adjudication in Stage 3 deserves more specification than a positive predictive value, because why discordance occurs is likely to be more informative than how often an alert is followed by an event. Table 3 proposes a classification for adjudicated discordant index times. Only the first three categories represent information the treating team needs; the remainder are properties of the record or of the models, and their frequencies define the engineering work that must precede any prospective trial. The distribution of categories, reported separately for positive and for negative discordance, should be a primary output of Stage 3 and should be re-estimated in Stage 4, since the mix will change with documentation practice and with model version. Negative discordance requires its own adjudication arm. Because there is often no objective event against which to score documented concern, its validation must rely on events at longer horizons, on the interventions that follow, and on the reviewer’s judgement of whether the concern was warranted by information that the structured record did not contain; this direction is arguably the more interesting of the two, since it is where the narrative can add what physiology cannot.

## 9. Limitations of This Proposal

Several objections deserve to be stated rather than deflected:

- The discordance score requires a well-calibrated physiological model as its reference. Where such a model does not exist for a given population or event definition, the discordance score inherits its deficiencies and may simply relabel physiological model error as narrative error.
- Nursing information is not confined to free text. Much of it is recorded in standardised form, as nursing diagnoses, assessments and interventions coded with standardised nursing terminologies, and it is frequently omitted from the structured datasets on which physiological models are built. Some apparent discordance may also reflect clinically relevant nursing information that is documented in standardised form but omitted from the structured comparator, rather than a true mismatch between narrative assessment and physiology [33]. Where standardised nursing data exist, they should be included in the structured comparator, and the content of that comparator should be reported with the score.
- Narrative and physiology are not independent. Clinicians document what the monitor shows, so the two components share information by construction, and the residual discordance may be small and noisy. Quantifying the mutual information between the two components should be a reported quantity, not an assumption (Section 3.3 and Table 2).
- Negative discordance, documented concern exceeding measured risk, is arguably the more clinically interesting direction, since it may encode bedside observation that never reaches the structured record. It is also the harder direction to validate, because there is often no objective event against which to score it; Section 8 proposes a separate adjudication arm for this direction.
- Documentation culture varies substantially between health systems, specialties and languages, and the sentiment literature is dominated by two North American datasets. Transportability should be treated as an open empirical question rather than an implementation detail.
- Finally, the intervention this tool implies, a prompt to re-examine a patient, is weakly specified. If prompted review does not change management, no improvement in discrimination will produce a change in outcome.

## 10. Conclusions

Clinical sentiment analysis has spent a decade demonstrating that the tone of clinical notes correlates with death. It does, and the correlation adds almost nothing to what severity scores already tell us. The information in the narrative is not prognostic redundancy; it is a record of the treating team’s documented assessment, at a specific time, of where the patient is going. That assessment becomes informative precisely when it diverges from what the data show, whatever the reason for the divergence proves to be.

We have proposed that the field adopt the narrative–physiology discordance score as its primary target: a signed, calibrated, uncertainty-bounded difference between two probabilities of the same short-horizon deterioration event, estimated from the documented assessment with transcribed physiology masked, computed at fixed index times, audited for leakage and copy-forward, adjudicated for cause, evaluated by decision curve rather than by AUROC alone, and thresholded against an alert budget agreed with the unit before the model is built. A system built this way will not tell the intensivist what the patient is, and until it has been adjudicated prospectively, it will not tell the team that anything has been missed. It will tell the team which parts of the record no longer agree with each other, and leave the interpretation where it belongs.

## Figures and Tables

**Table 1:** Model outputs, the quantity each estimates and the display elements required for a clinician to audit the output at the point of care. All three outputs share a single pre-specified event definition and forecast horizon.

Output	What It Estimates	Clinical Value	Mandatory Display Elements
Narrative trajectory score	Probability of the pre-specified deterioration event within horizon h, conditioned on text only	Detects a shift in the documented assessment before it reaches a formal escalation	Trend over time; source note spans; author role and grade (as provenance, not as predictors); role-specific scores where roles disagree; note timestamp and authoring modality
Physiological trajectory score	Probability of the same event within the same horizon, conditioned on time-aligned structured data only	Captures worsening organ dysfunction and rising treatment intensity	Vital-sign trends; vasopressor dose and rate of change; lactate; respiratory support; renal markers; fluid balance
Narrative–physiology discordance score	Signed logit difference between the two, with an uncertainty interval	Identifies patients warranting renewed review rather than automatic treatment; a record-level inconsistency signal, not a diagnosis	Direction and magnitude of mismatch; uncertainty interval; persistence across index times; time since last note; evidence spans; flagged data artefacts; abstention status

**Table 2:** Minimum reporting specification for studies of narrative–physiology discordance, aligned with TRIPOD + AI [16] and DECIDE-AI [17].

Element	Requirement	Rationale
Index time	Exact definition of when the prediction is made and how data availability is determined	Prevents implicit use of future information
Forecast horizon	Pre-specified (e.g., 6, 12 or 24 h), justified by the workflow the alert is meant to serve	A horizon shorter than the response time is unactionable
Outcome definition	Objective, adjudicable deterioration event, not mortality alone; physiologically anchored where possible, with intervention-defined events reported separately (Section 3.2)	Mortality is a distal proxy dominated by severity scores
Calibration	Calibration-in-the-large, calibration slope, flexible calibration plot, Brier score, reported separately for both components and the difference	A difference of two probabilities is only interpretable if both are calibrated
Shared information	Correlation or mutual information between the narrative and physiological scores; incremental contribution of the narrative score conditional on the physiological score; the masking rule applied to transcribed physiology	Bounds the information the difference can carry and separates assessment from transcribed physiology (Section 3.3)
Class-imbalance metrics	Precision–recall curve and area, positive predictive value at the deployed threshold	AUROC is optimistic when events are uncommon
Clinical utility	Decision-curve analysis with net benefit across the plausible threshold range	Accuracy metrics do not express clinical consequence
Comparators	Structured-data model alone; narrative model alone, masked and unmasked; jointly trained multimodal model; SOFA or early warning score; clinician-triggered review	Incremental value cannot be claimed without the relevant baseline; the jointly trained model quantifies the discrimination forgone for decomposability
Dynamic evaluation	Landmark analysis at repeated time points, not one prediction per admission	Single-prediction evaluation conceals time-dependent failure
Leakage audit	Pre-specified removal of decision-adjacent language occurring after index time; 24 h and 48 h truncation sensitivity analyses reported alongside the untruncated analysis (Section 4.1)	Outcome-adjacent text is the dominant source of inflated performance
Provenance controls	Text-similarity and templating metrics; note-signature latency; abstention rate	Copied text misdates the narrative signal
Uncertainty	Prediction intervals for both components and for the difference; alert eligibility defined on the interval (Section 3.4); an explicit abstention mechanism for sparse, contradictory, copied or out-of-distribution input, with the abstention rate and the event rate among abstentions reported	A confident wrong alert and an unqualified silence both carry cost; abstention makes uncertainty visible, and its rate must be reported so that missed events are counted rather than hidden
Adjudication	Clinician classification of a stratified sample of discordant index times using a pre-specified taxonomy (Table 3), reported separately for positive and negative discordance	Why discordance occurs is more informative than its positive predictive value alone
Transportability	External validation in a health system with different documentation culture and language	Sentiment is culture and language bound; drift is expected [18]

**Table 3:** Proposed adjudication taxonomy for discordant index times. Expected direction is the sign of *D*(*t*) with which each category is expected to be associated most often.

Category	Definition	Expected Direction	Implication
Unrecognised physiological deterioration	Structured data show deterioration that no contemporaneous note acknowledges, and review confirms that it was clinically real	Positive	The signal the framework is designed to surface
Recognised but undocumented deterioration	The team was aware and acting, but the note had not yet been written or updated	Positive	Documentation timeliness rather than a clinical miss; informs alert timing
Appropriate concern absent from structured data	Documented concern reflects bedside information (gestalt, work of breathing, perfusion, response to treatment) that the structured record cannot represent, and review judges it warranted	Negative	The signal negative discordance is meant to capture; may precede structured change
Documentation lag or expected modality difference	New structured data arrived after the last note and would have been incorporated at the next documentation opportunity	Positive, transient	Expected difference; resolves with the next note; supports the persistence criterion (Section 3.4)
Copy-forward artefact	The narrative reflects a prior state carried forward without re-assessment	Either	Provenance failure; should have triggered abstention
Structured-data artefact	Damped arterial line, erroneous laboratory value, mis-timed or mis-mapped data	Positive	Data-quality failure; should be flagged, not alerted
Model error, narrative component	The narrative model misreads the note, for example, through negation, end-of-life language or template text	Either	Model development target
Model error, physiological component	The physiological model is miscalibrated or out of distribution for this patient	Either	Model development target
Indeterminate	Reviewers cannot classify the case with the information available	Either	Reported, not discarded; its rate bounds the interpretability of the score

**Table 4:** Staged research agenda with exit criteria. No stage should be skipped; in particular, an interventional trial should not be considered before completion of Stage 4.

Stage	Objective	Design	Exit Criterion
1	Label infrastructure	Extend clinician-annotated ICU sentiment corpora [13] to multiple centres and languages, with span-level annotation and documented inter-annotator agreement	Publicly available multi-centre corpus with agreement statistics reported by note type and author role
2	Construct validation	Establish that the narrative trajectory score predicts short-horizon deterioration independently of SOFA and treatment intensity, at fixed index times, with full leakage and provenance audit	Calibrated narrative model with demonstrable incremental net benefit over a structured-data comparator in decision-curve analysis
3	Discordance characterisation	Retrospective multi-cohort study describing the distribution, persistence and antecedents of positive and negative discordance, with clinician adjudication of a stratified sample using the taxonomy in Table 3, and a separate arm for negative discordance adjudicated against subsequent events at longer horizons and against bedside information absent from the structured record	A defined discordance threshold with an adjudicated positive predictive value and a reported distribution of adjudication categories for positive and for negative discordance
4	Prospective silent trial	Deploy without clinician-visible output; measure alert frequency, positive predictive value, lead time relative to clinician-initiated escalation and subgroup performance	Alert rate within the pre-agreed budget and lead time exceeding usual care before any interventional trial is contemplated

## Data Availability

No new data were created or analyzed in this study. Data sharing is not applicable to this article.

## Abbreviations

APACHE, Acute Physiology and Chronic Health Evaluation; AUROC, area under the receiver operating characteristic curve; EHR, electronic health record; ICU, intensive care unit; MIMIC, Medical Information Mart for Intensive Care; SAPS, Simplified Acute Physiology Score; SOFA, Sequential Organ Failure Assessment.
